# A case series shows a relation between intracochlear electric field distribution and vestibular co-stimulation with cochlear implants

**DOI:** 10.1038/s41598-025-00887-6

**Published:** 2025-05-08

**Authors:** Luise Wagner, Torsten Rahne, Stefan K. Plontke, Laura Fröhlich

**Affiliations:** 1 Department of Otorhinolaryngology, Head & Neck Surgery, Martin Luther University Halle-Wittenberg, University Medicine Halle (Saale), Halle (Saale), Germany; 2 Present Address: Department of Otolaryngology, Head and Neck Surgery, University Hospital Bonn, Bonn, Germany

**Keywords:** e-VEMPs, Cochlear implant, Transimpedance matrix, Co-stimulation, Neurophysiology, Cochlea, Vestibuloocular reflex

## Abstract

The objective of this study was to investigate the relation between the electric field distribution within the cochlea during cochlear implant stimulation and the electrical vestibular co-stimulation measured by vestibular evoked myogenic potentials (e-VEMPs). Measurements were done in adult Nucleus cochlear implant (CI) users with perimodiolar electrode arrays. The electric field distribution within the cochlea was determined by Transimpedance Matrices recorded for all participants with a pulse width of 25 µs and a current level of 110 CL. Study measurements were conducted in 25 ears of 24 participants. In 10 participants, e-VEMPs could be elicited (40%). The occurrence of e-VEMPs stimulated by the cochlear implant was correlated with the magnitude of a corrected transimpedance at the most basal electrode. Since the results also suggest that there are patients with vestibular co-stimulation already present at their everyday CI stimulation level, this needs to be taken into account by audiologists creating programming maps for CIs, e.g. by deactivation of basal electrode contacts, if dizziness occurs during CI stimulation.

## Introduction

There are several studies investigating the electrical stimulation of the vestibular system^1–3^. In 1982 Eisenberg et al.^4^ already investigated the improvement of postural stability through the activation of cochlear implants. The effect of cochlear implantation on vestibular evoked myogenic potentials is described in different studies^5–8^. Additionally, co-stimulation during cochlear implant (CI) stimulation has been described in this context^9,10^. A recent study by Fröhlich et al.^11^ investigated the influence of CI stimulation parameters on the occurrence of e-VEMPs (electrically vestibular evoked myogenic potentials) in Nucleus CI users. It was shown that high stimulation levels and monopolar stimulation at basal CI electrode contacts increased the probability of evoking e-VEMPs and thus vestibular co-stimulation.

The stimulation of a CI electrode contact does not lead to a focused excitation of neurons but rather results in a spread of current within the cochlea. This can be measured with different tools. Spread of neural excitation (SOE) measurements have shown co-stimulation of neurons associated with other frequency areas and electrode contacts^12,13^. Spread of the electric field – without information about neural excitation – can be assessed by using the transimpedance matrix (TIM) measurement for Nucleus cochlear implants (Cochlear Ltd., Sydney, Australia). Other manufacturers refer to this measurement of spread of electric field as “electric field imaging” (EFI – Advanced Bionics, Stäfa, Switzerland, e.g^14^). or voltage matrix of the impedance and field telemetry (Med-El, Innsbruck, Austria, e.g^15^). A general and incorporating name for these tools is “Stimulation-Current-Induced Non-Stimulating Electrode Voltage recordings” (SCINSEVs)^16^. Here, we focus on TIM which shows transimpedances for 21 electrodes per each stimulating electrode in the cochlea. The stimulation current at one electrode is constant and the voltage at each other electrode on the array within the cochlea can be determined using telemetry (for a detailed description see^17^).

For CI stimulation, it is possible to reference the active electrode to all other passively connected electrodes inside the cochlea (common ground mode). However, a monopolar stimulation using extracochlear electrodes is widely used. For Nucleus cochlear implants, two extracochlear reference electrodes can be used. One contact is at the end of an extracochlear wire which is usually placed below the musculus temporalis^18^ during surgery. Another contact is positioned on the implant housing. Since they are positioned outside the cochlea, it can be assumed that also other structures besides the cochlear nerve might be electrically stimulated. One example is facial nerve co-stimulation as a side effect that can be seen during electric stimulation in some CI users^19,20^. Studies have shown that vestibular co-stimulation in CI users can be detected by the recording of electrically elicited vestibular evoked myogenic potentials (e-VEMPs)^2,3,21^. However, it is unknown which vestibular structures exactly are stimulated by the CI, i.e., the vestibular receptors (utricule and saccule) or the vestibular nerve, when e-VEMPs are elicited. The short e-VEMP latencies reported by Fröhlich et al.^11^ suggest direct stimulation of the vestibular nerve. Despite the unknown target structures the source of the electric current is the CI itself with the electric currents travelling along the cochlea. Thus, it can be assumed that the magnitude of the voltage, which is correlated to the magnitude of the transimpedance at the basal turn of the cochlea, could be a predictor for the occurrence of vestibular co-stimulation and the occurrence of e-VEMPS. In order to test this hypothesis, we investigated the relation between the TIM and presence of e-VEMPs in this study.

Ramos de Miguel et al. investigated co-stimulation of the vestibular organ by TIM measurements in four patients using a research implant consisting of an intracochlear and an intravestibular electrode array^22^. The authors concluded that there was no cross-stimulation from the cochlea to the vestibule or from the vestibular electrodes to the cochlea in TIM recordings.

Here, we used standard CIs with intracochlear electrode arrays and analysed TIMs in the basal part of the cochlea, hypothesizing that interindividual differences in the presence or absence of e-VEMPs are most pronounced at locations with a small distance to the vestibular receptors.

## Materials and methods

### Study design and participants

A prospective explorative study was conducted between June 2020 and December 2021 at a single tertiary referral centre. Inclusion criteria were being between 18 and 65 years old and having a Nucleus cochlear implant with a perimodiolar electrode array (Cochlear Ltd., Sydney, Australia) and full insertion. Patients with known vestibular disorders (e.g. vestibulopathy, Menière’s disease, vestibular migraine), cochleovestibular schwannoma, cochlear malformations, electrode displacement on postoperative imaging, and cochlear fibrosis or sclerosis were excluded. The study protocol was reviewed and approved by the ethics committee of the Martin Luther University Halle-Wittenberg (approval number: 2020-22) and performed in accordance with the relevant guidelines and regulations. Written informed consent was obtained from all participants.

### Experimental setup and procedures

All measurements were conducted in a soundproof and electromagnetically shielded booth suitable for audiological and electrophysiological measurements. CI stimulation was controlled using Custom Sound EP software (version 6.0, Cochlear Ltd., Sydney, Australia). Transimpedance measurements were conducted in the respective module using biphasic pulses with a pulse width of 25 µs and a current level (CL) of 110. For stimulation, monopolar mode (MP1) was used and MP2 mode was used for recording. The time of recording was at the end of the first half of the biphasic pulse (T06). A stimulation at higher levels, which were used for e-VEMP stimulation, was not tolerated by all patients.

For the e-VEMP recordings, electric pulses were generated in the eABR module of Custom Sound EP software (version 6.0, Cochlear Ltd., Sydney, Australia). An external trigger signal was generated in the module and sent to the Eclipse (Interacoustics, Middlefart, Denmark) recording system via the CI programming pod. For all participants the same test audio processor (CP910, Cochlear, Sydney, Australia) was used for stimulus transmission to the implant. Electric tone bursts were composed of biphasic pulses (25 µs pulse duration, 7 µs interphase gap) with a burst/stimulation rate of 1000 Hz and burst duration of 3.057 ms (= 4 pulses at 1000 Hz) and presented at 8 Hz. The stimulation was done in monopolar mode between the housing electrode and the basal intracochlear electrode E3. For all participants, the maximum tolerable stimulation level (MTSL) at stimulating electrode E3 was determined by subjective loudness scaling. The stimulation for e-VEMP recording started at that level. The specific setup and procedure for e-VEMP recordings can be found in Fröhlich et al.^11^.

### Data analysis

The VEMP data were analysed according to the procedure described by Fröhlich et al.^11^. For this study, participants were divided into two groups – one group of participants with recordable e-oVEMPs and/or e-cVEMPs (e-VEMP) and one group of participants without recordable e-VEMPs within the MTSL (no e-VEMP).

The TIM data for all patients were exported and plotted with python (version 3.8) as single matrix plots. The maximum for plotting was set to 2 kOhm. The recorded transimpedance line plot for stimulation at electrode E3 was plotted for both groups. The transimpedances at basal electrodes E2 and E1 for stimulation at electrode E3 were further analysed and distributions are shown as boxplots. *T*-tests were calculated for the comparison of group means and Bonferroni correction was applied for multiple comparisons.

As the results by Fröhlich et al.^11^ showed a strong effect of the maximum tolerable stimulation level on the occurrence of e-VEMPs, further analysis was performed to consider these individual tolerable levels of patients. The maximum tolerable comfort level (MCL) was measured by subjective loudness scaling, i.e., asking the patient when the stimulus used for e-VEMP measurements could not be tolerated to be any louder. For one patient, the TIM measurement was repeated with different stimulation levels (110 CL to 150 CL in 10 CL steps) to examine the change of transimpedance as a function of the stimulation level. Line graphs for all stimulation levels were plotted. A correction factor was derived from the increase of the transimpedance for an increase of stimulation by 10 CL. The correction was then used to calculate an estimated transimpedance at the maximum tolerable stimulation level for each patient. Corrected group means were plotted again as line graphs to compare the two groups with respect to the maximum tolerable stimulation level.

## Results

The study included recordings in 25 CIs of 24 patients between 23 and 63 years of age. The mean age was 50.8 years (SD: 12.2 years). Further information about the study sample can be found in Table 1.

**Fig. 1:**
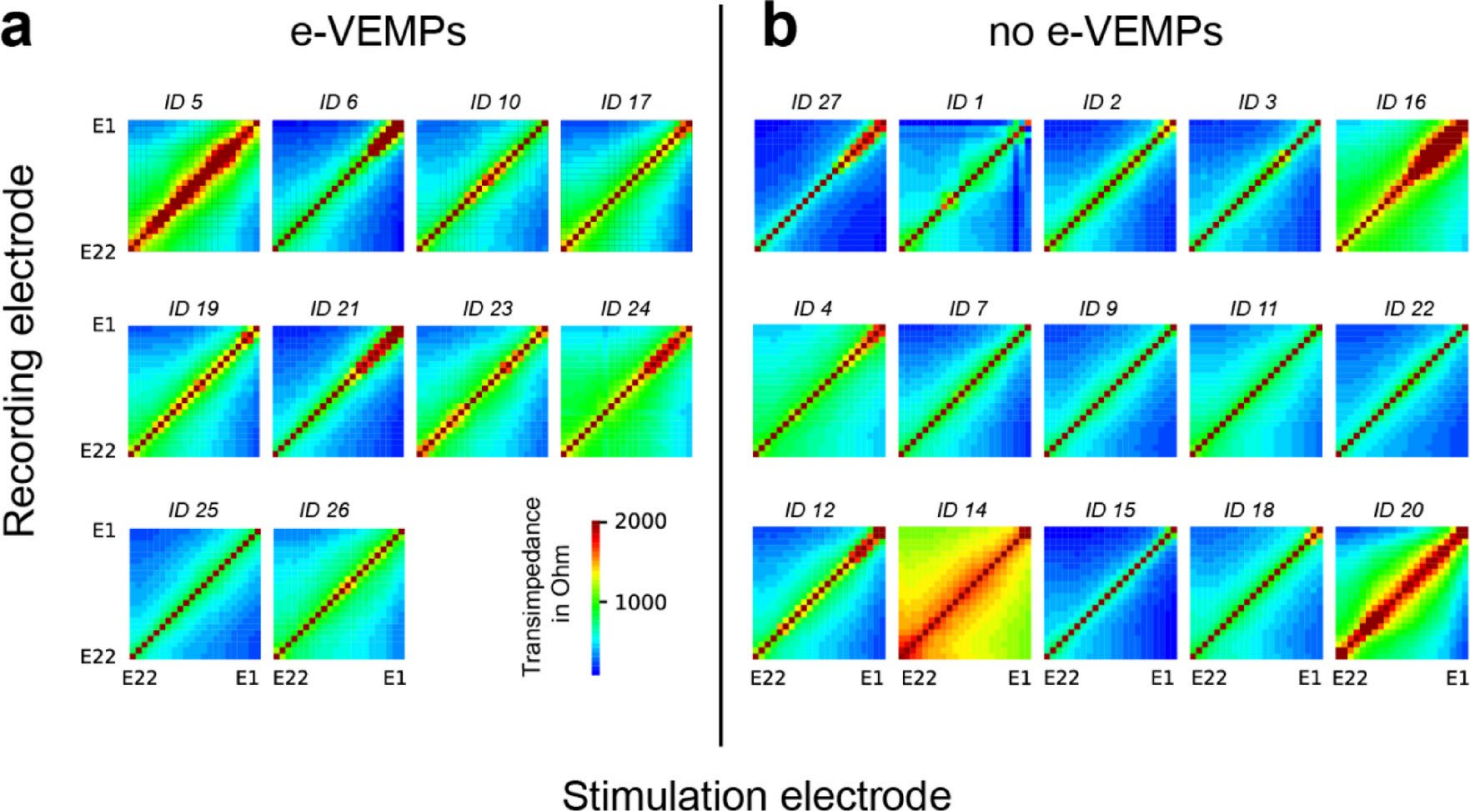
Overview of TIM for all participants. (**a**): TIMs of patients with evocable e-VEMPs. (**b**) : without measurable e-VEMPs. All TIMs were measured with 25 µs pulse width and 110 CL pulse amplitude.

All individual TIMs are shown in Fig. 1. Visual comparison revealed no obvious difference concerning the width (in respect to the diagonal) or shape of the electric field between the two groups. There were wide (e.g. ID 5, 24, 14, 16, 20) and narrow (e.g. ID 6, 25, 27, 3, 15) distributions of the electric field in both groups.

**Fig. 2:**
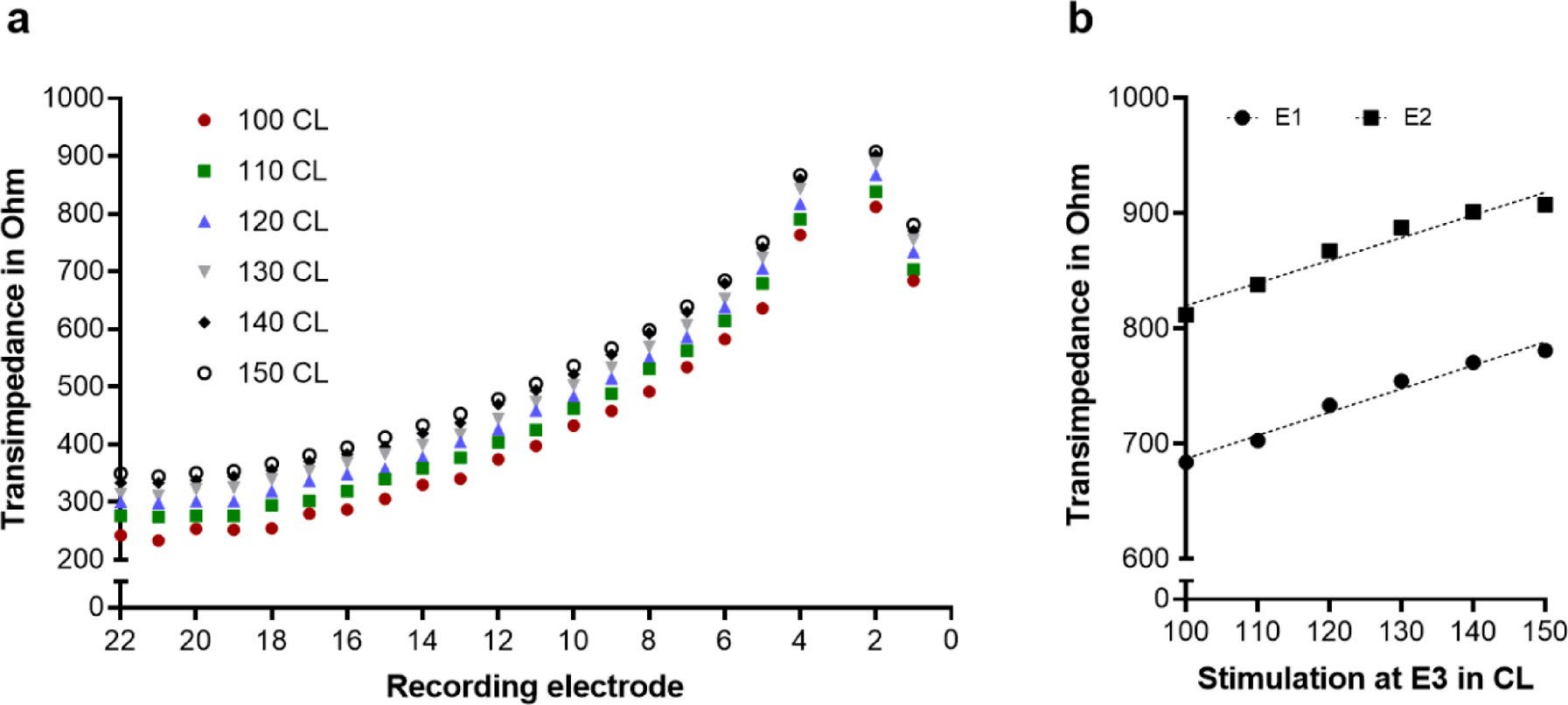
Transimpedance depending on stimulation level. (**a**): Transimpedances for stimulation at electrode E3 with increasing stimulation level from 100 CL up to 150 CL for one participant (eVEMP_NM_23). (**b**): Transimpedance at electrode E1 and electrode E2 as function of stimulation level at electrode E3. Dotted lines show linear regression. CL: current level.

Figure 2A shows the transimpedances for stimulation at electrode 3 and increasing stimulation level for one participant. An increase of 10 CL led to an increase in transimpedance for a single electrode contact of 10 to 20 Ohm. In Fig. 2B the relation between increasing stimulation level and increasing transimpedance is shown. A regression analysis showed a linear relation with a slope of 2.03 Ohms per CL (SD: 0.16; 95% CI: 1.59 to 2.46) at electrode 1 and 1.97 Ohms per CL (SD: 0.22; 95% CI: 1.36 to 2.57) at electrode 2. Thus, a correction of 2 Ohm per 1 CL was applied for further analyses.

**Fig. 3:**
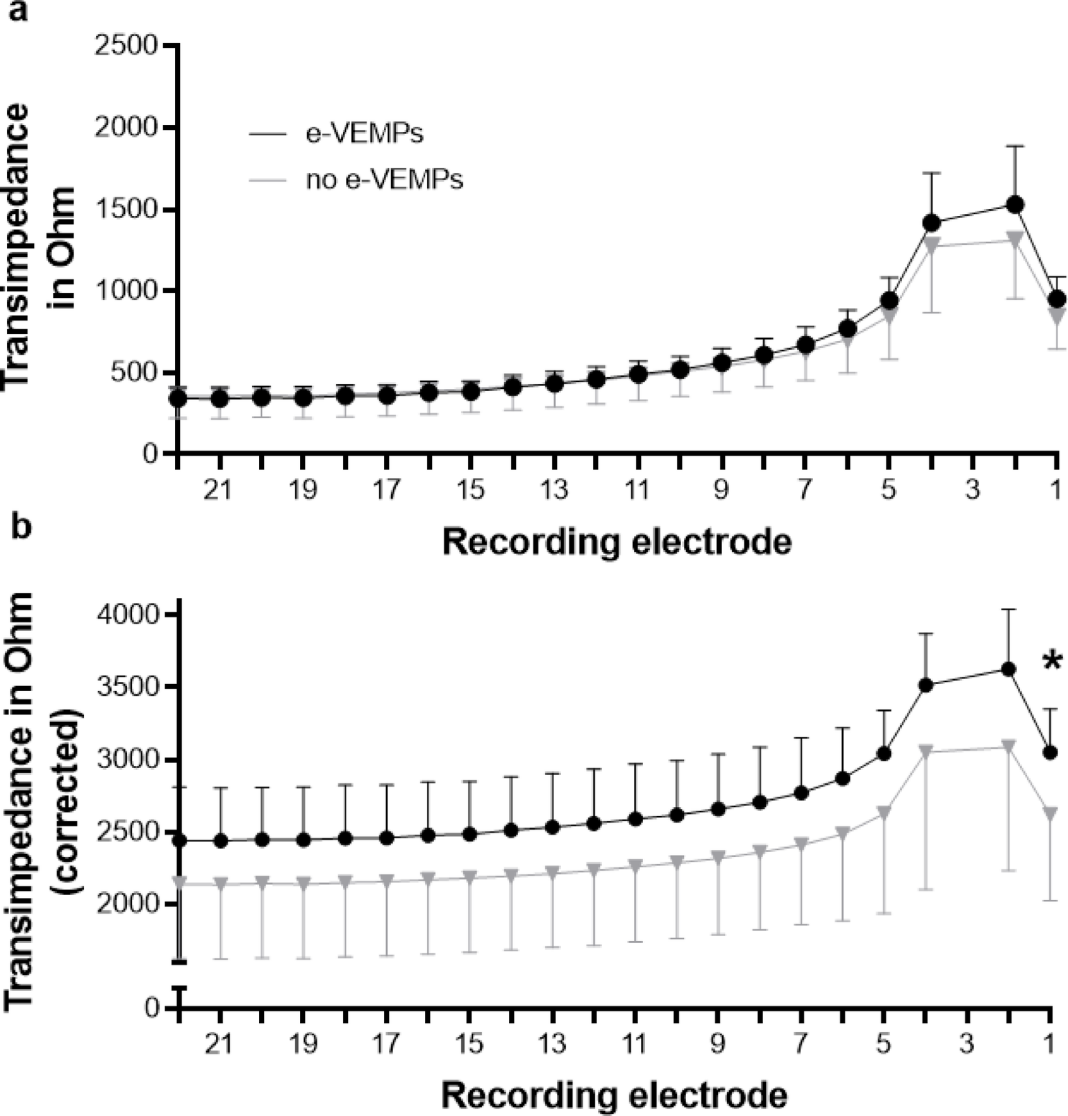
Corrected transimpedances. Transimpedances in line plots with stimulation at electrode E3 (mean and standard deviation). (**a**): uncorrected and (**b**): corrected transimpedances according to the maximum tolerable stimulation level. * < 0.05.

**Fig. 4:**
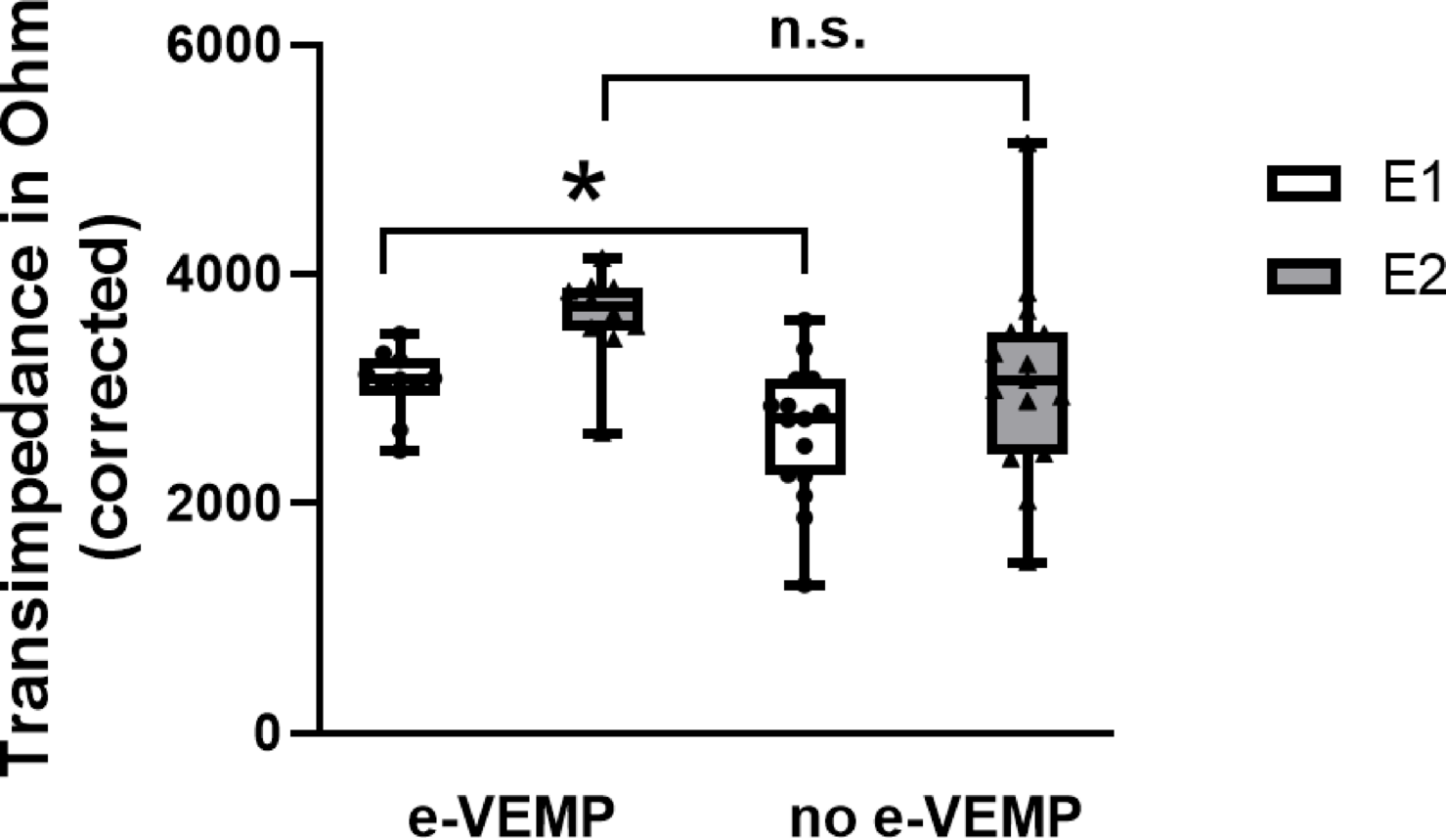
Comparing e-VEMP and no e-VEMP group. Transimpedance after correction according to maximum tolerable stimulation levels recorded at electrode E1 (white) and E2 (grey). For stimulation of electrode E3, a significant difference was observed between both groups of participants for E1. Larger transimpedance and hence larger voltage, can be found for the e-VEMP group. In the Boxplots, median, minimum and maximum are plotted. * significant difference, n.s. not significant.

Figure 3A shows the uncorrected line plots of the transimpedance at electrode 3 for both groups with all TIMs measured at 110 CL. Higher transimpedances in the basal part of the cochlea were observed for the e-VEMP group compared to the no e-VEMP group. In the e-VEMP group, the transimpedances were 1530 ± 500 Ohm at electrode 2 and 952 ± 190 Ohm at electrode 1. In the no e-VEMP group the transimpedances were 1308 ± 640 Ohm and 840 ± 355 Ohm, respectively. The differences were not statistically significant (both *p*s = 0.37, Fig. 3). Figure 3B shows the line plots of the TIM corrected for individual maximum tolerable stimulation level by 2 Ohm / 1 CL. After the correction of TIM values, the differences in transimpedance values of electrode 1 between the two groups were larger. At electrode 1, transimpedance in the e-VEMP group was 3052 ± 300 Ohm compared to 2620 ± 597 Ohm (*p* = 0.046) in the no e-VEMP group. At electrode 2, the difference between e-VEMP and no e-VEMP group was not statistically significant (*p* = 0.076). The individual data for the transimpedance at electrode 1 and 2 are shown in Fig. 4.

**Table 1:** Demographic data of participants. MTSL – maximum tolerable stimulation level, Y – years, M – months, f – female, m – male, r – right, l – left, C – cochleostomy, R – round window.

Subject ID	Implant type	Duration of use	Age in Y	Sex	Test side	MTSL	Threshold of e-VEMP	Insertion
e-VEMP group								
eVEMP_ 05	CI24R	15 Y	55	f	r	220 CL	180 CL	C
eVEMP_ 06	CI24RE	7 Y	59	f	r	200 CL	190 CL	R
eVEMP_ 10	CI24RE	12 Y	56	f	r	235 CL	185 CL	C
eVEMP_ 17	CI632	4 M	49	f	r	220 CL	220 CL	R
eVEMP_ 19	CI512	6 Y	58	m	l	220 CL	220 CL	R
eVEMP_ 21	CI632	1 Y	35	m	l	190 CL	175 CL	R
eVEMP_ 23	CI24RE	8 Y	24	f	r	220 CL	200 CL	R
eVEMP_ 24	CI512	5 Y	63	f	r	215 CL	215 CL	R
eVEMP_ 25	CI632	1 Y	23	f	r	240 CL	200 CL	R
eVEMP_ 26	CI24RE	13 Y	63	f	r	190 CL	190 CL	C
Average e-VEMP group		6.8 +/- 5.2	48.5 +/- 15.5			215 +/- 17	200 +/- 15	
no e-VEMP group								
eVEMP_ 01	CI512	8 Y	59	m	r	225 CL	–	R
eVEMP_ 02	CI512	3 Y	42	f	l	200 CL	–	R
eVEMP_03	CI24RE(CA)	7 Y	60	m	l	195 CL	–	R
eVEMP_04	CI512	3 Y	61	f	r	210 CL	–	R
eVEMP_07	CI632	2 M	36	m	l	215 CL	–	R
eVEMP_09	CI532	1 Y	60	f	l	145 CL	–	R
eVEMP_11	CI532	2 Y	47	f	l	215 CL	–	R
eVEMP_12	CI532	2 Y	60	f	l	205 CL	–	R
eVEMP_14	CI532	3 Y	43	f	l	205 CL	–	R
eVEMP_15	CI632	1 Y	55	f	r	190 CL	–	R
eVEMP_18	CI632	1 Y	60	f	l	185 CL	–	R
eVEMP_20	CI512	4 Y	35	m	r	185 CL	–	C
eVEMP_22	CI632	1 Y	58	f	r	175 CL	–	R
eVEMP_16	CI532	2 Y	63	f	l	210 CL	–	R
eVEMP_27	CI532	2 Y	47	m	r	225 CL	–	R
Average no e-VEMP group		2.7 +/- 2.2	52.4 +/-9.7			199 +/- 21		
Average all		4.3 +/- 4.2	50.8 +/- 12.2			205 +/- 21		

## Discussion

This study is the first that looked at the occurrence of vestibular co-stimulation in CI users as indicated by the presence of e-VEMPs and that analysed the intracochlear electric field distribution in these patients to investigate the role of current spread. None of the participating patients reported any vestibular problems.

In this study, visual inspection of all single TIMs (Fig. 1) did not show a difference in width or shape between the e-VEMP group, in which patients with o- and c-VEMPs are combined, and the non e-VEMPs group. A more detailed look at the stimulation of electrode E3, which was the electrode contact that evoked the e-VEMPS in this study, revealed differences between transimpedances in the basal cochlear region of trial subjects in the e-VEMP and the no e-VEMP group. The mean transimpedance, i.e., voltage, was higher in the e-VEMP group than in the no e-VEMP group. However, the trend was not statistically significant, possibly due to the small sample size and standard stimulation current of 110 CL used for TIM recordings.

One of the major challenges in this study was to consider the influence of interindividual differences of maximum tolerable stimulation levels. The question arose whether an increase of stimulation level, if tolerated, can lead to a vestibular co-stimulation and whether this was also reflected by the electric field distribution. E-VEMP response rates increased with increasing stimulation level according to Fröhlich et al.^11^. Thus, some patients may have been classified into the no e-VEMP group because of insufficient stimulation level, even though the electric field distribution could be rather large, which would facilitate co-stimulation. The measurement of the transimpedance was originally done in this study using a fixed stimulation level of 110 CL to keep it comparable and to possibly find a systematic difference in field distribution between groups. Analysing the effect of increasing the current level (CL) on the transimpedance measurements in a single subject, we found an increase of 2 Ohm per 1 CL (Fig. 2). An increase in stimulation level only resulted in a shift in the transimpedance but not in a change of the distribution of the electric field. The application of this correction of 2 Ohm per CL to the TIM data revealed a significantly higher transimpedance, i.e. voltage, at basal electrode contact E1 in the e-VEMP group as compared to the no e-VEMP group. Thus, a larger spread of electric field towards basal electrodes was associated with the occurrence of e-VEMPs, which supports the theory that electric current spread is responsible for co-stimulation.

Our data are consistent with the results of Ramos de Miguel et al.^22^ although we came to a different conclusion. We would also interpret their data in Fig. 1 of^19^ as a current flow between vestibular electrodes and basal cochlear electrodes in some cases (except the right bottom one). At least electrode 4, which is the most basal one in their implant, shows crosstalk. This is in line with our results.

Different factors might have an influence on the distribution of the electric field and need to be considered. The influence of the surgical technique and the implant type were not analysed in this study. A cochleostomy was performed 3 times in the e-VEMP group and only once in the no e-VEMP group (Table 1). The mean duration of use was longer and thus the implant model was “older” in the e-VEMP group (Table 1). This inhomogeneity is a limitation of the study and needs further investigation in future studies.

Since the intracochlear electric field depends on many factors which are not fully understood and are different in every CI patient, the risk for unintended vestibular co-stimulation is an individual risk. Among other factors, especially stimulation level, individual current spread may play a role. In this study sample, one patient’s e-VEMP threshold was even within the everyday current level range (cochlear stimulation). In clinical routine CI fitting, it is therefore necessary to keep in mind that vestibular co-stimulation is possible and might have clinical relevance. Deactivation of basal electrode contacts could help in cases when dizziness due to CI use is reported by patients.

At that point it is not possible to use the TIM measure as a predictor for e-VEMP occurrence but we were able to show a correlation between the technical measure and the vestibular co-stimulation on group level. We are of the opinion that it is valuable to investigate this correlation further and analyse the effect of more influencing factors in further studies to develop a predictor in the future.

## Data Availability

The data of this research project are available on reasonable request from the corresponding author via mail contact (luise.wagner@uk-halle.de).
